# Mitigating burnout and suicidal ideation in the Brazilian health care workforce: the role of workplace support during COVID-19

**DOI:** 10.47626/1679-4435-2024-1290

**Published:** 2025-01-31

**Authors:** Carolina Meira Moser, Bárbara Tietbohl-Santos, Ana Margareth Siqueira Bassols, Pricilla Braga Laskoski, Simone Hauck

**Affiliations:** 1 Graduate Program in Psychiatry and Behavioral Sciences, Universidade Federal do Rio Grande do Sul, Porto Alegre, RS, Brazil.; 2 Psychodynamic Psychiatry Research Laboratory, Hospital de Clínicas de Porto Alegre, Porto Alegre, RS, Brazil; 3 Division of Health Promotion, Department of Health, Universidade Federal do Rio Grande do Sul, Porto Alegre, RS, Brazil.; 4 Deutschen Akademie für Psychoanalyse, Münchner Lehr- und Forschungsinstitut, München, Deutschland.

**Keywords:** health personnel, burnout, professional, working conditions, COVID-19, suicidal ideation, pessoal de saúde, esgotamento profissional, condições de trabalho, covid-19, ideação suicida

## Abstract

**Introduction:**

The mental health of health care workers has become a major concern, especially in the context of the COVID-19 pandemic. Identifying associated factors that could be targeted for prevention and specific interventions is crucial.

**Objectives:**

To investigate burnout, suicidal ideation, and associated factors among Brazilian health care workers during the pandemic.

**Methods:**

A cross-sectional web-based survey was conducted from May 22 to June 22, 2020. The Copenhagen Burnout Inventory was used to assess three dimensions of burnout (personal, work-related, and client-related). Individual and occupational data were also evaluated.

**Results:**

Among 844 participants (81% female, age 41.9 ± 10.9 years), clinically relevant burnout rates were 54.6% according to the Copenhagen Burnout Inventory personal dimension, with an 8.3% incidence of suicidal ideation in the last month. Workplace support exhibited the strongest association with all three burnout dimensions (β = -0.29-0.37; p < 0.001) and suicidal ideation (Exp[b] = 0.95; p = 0.002), emerging as a crucial protective factor, even when adjusting for other variables. Older age, higher household income, and regular physical exercise also emerged as protective factors against burnout, but not against suicidal ideation. Female gender, direct involvement in care of COVID-19 patients, longer working hours, and self-perceived high-risk status for COVID were risk factors solely for burnout. Childhood trauma and a history of psychiatric diagnosis were associated with both burnout and suicidal ideation.

**Conclusions:**

Fostering a supportive work environment could prove to be an efective strategy to mitigate mental health risks among health care workers in response to chronic stress, even in vulnerable contexts such as major health crises.

## Abstract (pt)

**Introdução:**

A saúde mental dos profissionais de saúde tornou-se uma preocupação importante, especialmente durante a pandemia da covid-19. Identificar fatores associados para desenvolver estratégias efetivas é fundamental.

**Objetivos:**

Investigar *burnout,* ideação suicida e fatores associados em trabalhadores da saúde brasileiros durante a pandemia.

**Métodos:**

Pesquisa *online* realizada de 22 de maio a 22 de junho de 2020. *Burnout* pessoal, *burnout* relacionado ao trabalho e *burnout* relacionado ao paciente foram avaliados através do Inventário de *Burnout* de Copenhagen, juntamente a dados individuais e ocupacionais.

**Resultados:**

Foram incluídos 844 participantes (81% do sexo feminino, 41,9 ± 10,9 anos): 54,6% apresentaram níveis clinicamente significativos de *burnout* segundo a dimensão pessoal do Inventário de *Burnout* de Copenhagen e 8,3% reportaram ideação suicida no mês anterior. A percepção de suporte no local de trabalho apresentou a associação mais forte com as três dimensões de *burnout* (ß = -0,29-0,37; p < 0,001) e ideação suicida [Exp(b) = 0,95; p = 0,002], emergindo como um fator de proteção crucial, mesmo após controle para outras variáveis. Idade mais avançada, maior renda familiar e exercício físico regular também surgiram como fatores protetores para o *burnout,* mas não para a ideação suicida. Gênero feminino, assistência direta a pacientes com covid-19, jornadas de trabalho mais longas e autopercepção de alto risco para covid foram fatores de risco exclusivamente para o *burnout.* Trauma infantil e histórico de diagnóstico psiquiátrico foram associados tanto a *burnout* quanto à ideação suicida.

**Conclusões:**

A promoção de um ambiente de trabalho suportivo apresenta-se como potencial estratégia efetiva para mitigar os riscos do estresse crônico à saúde mental dos profissionais de saúde, mesmo em contextos vulneráveis, como o de grandes crises de saúde.

## INTRODUCTION

Occupational burnout is a condition which develops in response to chronic stress in the workplace, generating physical, emotional, and mental exhaustion.^1^ It can lead to adverse consequences, such as depression, substance use, anxiety, and an increased risk of suicide.^2,3^ In fact, in a longitudinal study of United States medical students, burnout was predictive of suicidal ideation even when depressive symptoms were not present.^4^ Moreover, the impact of burnout on health care systems has been a major concern, as it has been associated with higher institutional costs as well as with reduced empathy, suboptimal patient care, higher frequency of medical errors, and absenteeism in health care workers (HCWs).^5^

During the COVID-19 pandemic, the already widespread issue of high burnout rates among HCWs became an even more pressing concern due to the unprecedented occupational risks and other changes associated with the onset of the pandemic.^6,7,8^ According to a recent systematic review and meta-analysis,^9^ burnout was mainly associated with contact with patients, supply shortages, and having household activities affected by work. Studies have generally found that the emergence of COVID-19 heightened existing stressors and risk factors,^6,8^ such as increased working hours, sleep deprivation, work-life imbalance, having children, and being female. These factors have also been found to impact rates of suicidal ideation.^10,11^ On the other hand, a protective effect of organizational support on work stress and mental health has been observed.^12^

Nevertheless, more comprehensive studies regarding burnout and suicidal ideation among HCWs are scarce, particularly in low- and middle-income countries (LMICs).^13,14,15^ This gap may jeopardize the achievement of global objectives outlined in the World Health Organization (WHO) Comprehensive Mental Health Action Plan 2013-2030 and the Sustainable Development Goals, which aim to promote and protect mental health by transforming the environments that impact mental well-being.^16^

Given the heightened risk of mental health challenges during significant health crises,^11^ particularly in LMICs, where suicide rates are notably higher,^17^ it is essential to identify both risk factors and potential protective measures. These elements can then inform targeted interventions to mitigate the impact of such crises on vulnerable populations. A comprehensive understanding of these factors is essential for the development of effective strategies to mitigate the problem.^7^ Therefore, our study aims to explore both individual (e.g., socio-demographic, psychiatric history, lifestyle) and work-related (e.g., workload, direct care of COVID-19 patients, and work environment aspects such as leadership style, shared values, collaborative climate, and sense of belonging) factors associated with burnout and suicidal ideation in a sample of Brazilian HCWs during the COVID-19 pandemic.

## METHODS

### SETTINGS, DESIGN, AND PARTICIPANTS

This is a cross-sectional web-based survey conducted via an anonymous 95-item questionnaire deployed on the SurveyMonkey platform (http://www.surveymonkey.com). The survey was distributed through social media channels using a convenience snowball sample of Brazilian HCWs. The procedures and characteristics of the original sample are further described elsewhere.^18^ The sole exclusion criterion for the present study was being exclusively self-employed.

### DATA COLLECTION AND ETHICAL CONSIDERATIONS

Data were collected between May 22 and June 22, 2020, during the first peak of COVID-19 contagion in Brazil. The study protocol was approved by the Research Ethics Commitee of Hospital de Clínicas de Porto Alegre (Certificate of Ethical Review Submission: 30745020.5.0000.5327). All participants provided informed consent online. The investigation was carried out following the latest version of the Declaration of Helsinki and complied with Brazilian National Health Council Resolution 466/2012, which governs human subject research in the country.

### MEASURES

#### Individual variables

The online questionnaire included sociodemographic variables, lifestyle questions (practice of physical activity, sleep quality, and hours of sleep), workload (weekly work hours), workplaces, and years of professional experience as a HCW.

#### COVID-19-related questions

Involvement in care of COVID-19 patients was evaluated with the following question: “Have you provided professional assistance to individuals with a suspected and/or confirmed diagnosis of COVID-19?” Participants could choose between “Yes, in the direct care of these individuals,” “Yes, but not in the direct care of these individuals,” and “No.” If the participant answered “Yes, in the direct care of these individuals,” additional questions were applied. These questions included the frequency of access to personal protective equipment (PPE) (always, often, sometimes, rarely, or never), leaving home (one’s usual place of residence) to protect family members from contagion, and having been coerced into performing a professional activity which they did not feel qualified for or did not wish to carry out.

Participants also answered questions regarding testing positive for COVID-19 and whether they perceived themselves as belonging to a high-risk group for COVID-19 (i.e., being older than 65 or having morbid obesity, chronic pulmonary disease, severe heart disease, diabetes, immunosuppression, or other clinical condition that made respondents perceive themselves as belonging to a high-risk group for COVID-19).

#### Suicidal ideation and psychiatric antecedents

The presence of suicidal ideation was evaluated with the following question: “In the last month, have you wanted to kill yourself or had thoughts of killing yourself?” (Yes/No). Participants also responded to questions about whether they had ever received a psychiatric diagnosis (Yes/No) and if they had been a victim of physical, psychological, and/or sexual abuse during their childhood/adolescence (Yes/No).

#### Burnout

Self-perception of being in a state of burnout was assessed with the following question: “Do you consider yourself in burnout? (burnout is described by the WHO^19^ as the presence of feelings such as exhaustion or lack of energy, mental disconnection from work, or feelings of work-related negativism or cynicism that may be associated with reduced professional effectiveness).” Participants could choose between “Yes” or “No.”

Moreover, the Copenhagen Burnout Inventory (CBI) was used to evaluate burnout symptoms. The CBI was developed by Kristensen et al.^2^ and considers fatigue and exhaustion as the main constructs of burnout. It is a self-report 19-item questionnaire rated on a 5-point Likert scale. Each answer can range from 0 to 100: “Never/almost never” or “ To a very low degree” (0 points), “Seldom” or “ To a low degree” (25 points), “Sometimes” or “Somewhat” (50 points), “Often” or “ To a high degree” (75 points), and “Always” or “ To a very high degree” (100 points).

The CBI includes three sub-dimensions which should be used independently: Personal burnout (PB – the degree to which one perceives oneself to be physically and psychologically exhausted), Work-related burnout (WB – the degree to which physical and psychological exhaustion is perceived concerning work activities), and Client-related/patient-related burnout (CB – the level of exhaustion that one perceives as stemming from the professional relationship with clients/patients). The PB dimension (six items) may be applied to any subject, while the WB questions (seven items) assume that the respondent has paid work of some kind, and the CB dimension (six items) implies that the respondent works with people.

The CBI items in each subscale are summed and averaged to obtain the scores. The higher the scores, the higher the level of burnout. Differences of 5 points or more in total subscale scores are considered clinically significant according to the literature.^2^ Moreover, values equal to or greater than 50 are considered suggestive of high levels of burnout. When advisable, the PB score can be used as a measure of global burnout.^2^ A Brazilian Portuguese version has been validated in students^20^ and in HCWs.^21^

#### Work environment

The Work Environment Evaluation Instrument-7 (WEEI-7)^22^ was applied to evaluate organizational aspects such as the leadership style (e.g., fostering collaboration, emphatic listening, and atitudes towards employees), shared values, the presence of a collaborative climate, and the sense of belonging. It comprises seven items selected through a series of studies that assessed various aspects related to the work environment and its correlation with burnout symptoms among medical residents, as well as in a national sample of medical students and physicians in Brazil.^23,24,25^

It uses a five-point Likert scale, ranging from “totally false” (zero points) to “totally true” (four points). Total scores range from zero to 28 points; higher scores correspond to healthier and supportive work environments. The WEEI-7 showed sound validity and reliability in a sample of 2,537 Brazilian HCWs and medical students, with a unidimensional model that revealed an excellent fit to this data. All items loaded significantly on the unidimensional latent trait with factor loadings ranging from 0.583 to 0.869. McDonald’s omega was 0.89, indicating high internal consistency.^22^

### STATISTICAL ANALYSIS

Statistical analysis was performed using SPSS version 21. Histograms were used to evaluate the normality of the data. Descriptive analyses were reported as means, standard deviations (SD), or absolute and relative frequencies (n and %). Inferential statistics were selected according to the distribution of the data. Groups were compared with Student’s *t*-test or the Mann-Whitney *U* test as appropriate. The chi-square (χ^2^) test was used to compare categorical variables. Analysis of variance (ANOVA) with post-hoc Tukey’s test was applied to compare variables with more than two groups. Parametric analyses were reported using means and SD. Pearson’s or Spearman’s correlation coefficient (as appropriate) were used to test the strength and direction of correlations between continuous variables.

The presence of high levels of burnout (i.e., clinical burnout) according to the CBI (PB, WB, and CB domains with scores equal to or greater than 50) was reported as the percentage of cases screening positive for elevated burnout levels.

Hierarchical multivariate linear regression analysis was used to control for confounding factors associated with CBI scores. The blocks were built as follows: age, sex, and family income; 2) childhood trauma block (intermediate level); 3) work and lifestyle block. The criteria for including variables in the multivariate model were based on existing literature^8,9,26^ and the results obtained from bivariate analyses with the CBI scores. Variables with p < 0.10 were carried forward to the subsequent block. The regression or angular coefficient (B) was calculated with a 95%CI. This coefficient measures the effect on the outcome at each increase of one factor unit. Additionally, the standardized beta coefficient (β) was presented to compare the strength of the association between variables.

Hierarchical Poisson regression analysis was performed to control for confounding factors related to the occurrence of suicidal ideation in the last month. The blocks and the p < 0.10 criterion were the same used in the hierarchical multivariate linear regression analysis. The regression coefficient exponential [Exp(b)] was calculated, along with respective 95%CI, to establish the prevalence ratios.

Statistical significance was set at p < 0.05 and all tests were two-tailed.

## RESULTS

A total of 844 individuals met the inclusion criterion. The most frequent professional categories were physicians (36.4%, n = 307), nursing technicians (20.6%, n = 174), and nurses (16%, n = 135). Most participants were female (81%), white (74.9%), married or in a stable relationship (57.1%), residing in the Southern region of Brazil (62%), and had a household income above 5,000 BRL (61.1%). The mean age was 41.9 (± 10.9) years, the average length of professional experience was 15.3 (± 10.6) years, and the average hours worked weekly were 39.3 (± 17.5) (Table 1).

**Table 1:** Demographic, work-related, and lifestyle characteristics of the study participants

Variable	Total sample (n = 844)
Sex, n (%)	
Male	160 (19.0)
Female	684 (81.0)
Ethnicity, n (%)	
White	632 (74.9)
*Pardo* (mixed-race)	146 (17.3)
Black	46 (5.5)
Asian	11 (1.3)
Indigenous	4 (0.5)
Other	5 (0.6)
Marital status, n (%)	
Married/civil union	482 (57.1 )
Separated/divorced	104 (12.3)
	
Single, dating	129 (15.3)
Single, not dating	116 (13.7)
Widowed	13 (1.5)
Household income*, n (%)	
Up to 1,500 BRL	40 (4.7)
1,500 to 3,000 BRL	140 (16.6)
3,000 to 5,000 BRL	148 (17.5)
5,000 to 10,000 BRL	178 (21.1)
More than 10,000 BRL	338 (40.0)
Has children, n (%)	510 (60.4)
Workplace, n (%)	
University	95 (11.3)
Public hospital	349 (41.4)
Private hospital	234 (27.7)
Outpatient primary care setting	223 (26.4)
Outpatient mental health setting	45 (5.3)
Private office	231 (27.4)
Number of workplaces, median (P25-P75)	1 (1-2)
Care to COVID-19 patients, n (%)	
No	233 (27.6)
Yes, indirect	204 (24.2)
Yes, direct	407 (48.2)
Confirmed COVID-19 diagnosis	51 (6.0)
High-risk group for COVID-19	227 (26.9)
Physical activity	220 (26.1)
Self-perceived state of burnout	267 (31.6)
History of childhood abuse or trauma	250 (29.6)
History of psychiatric diagnosis	284 (33.6)
Suicidal ideation in the last month	70 (8.3)
Occupation, n (%)	
Physician	307 (36.4)
Nurse	135 (16.0)
Nursing technician	174 (20.6)
Psychologist	76 (9.0)
Other	152 (18.0)
Age, mean ± SD	41.9 ± 10.9
Years of professional experience, mean ± SD	15.3 ± 10.6
Hours of work/week, mean ± SD	39.3 ± 1 7 .5
Hours of sleep/day, mean ± SD	6.9 ± 1.6
CBI scores, mean ± SD	
PB	52.08 ± 20.45
WB	4 7.3 4 ± 20.03
CB	38.25 ± 24.02
WEEI-7 scores, mean ± SD	16.74 ± 6.84

CB = patient-related burnout; CBI = Copenhagen Burnout Inventory; PB = personal burnout; SD = standard deviation; WB = work-related burnout; WEEI-7 = Work Environment Evaluation Instrument-7.

* For the purposes of this study, 5.22 BRL = 1 USD.

Regarding lifestyle, 75.6% (n = 630) reported not engaging in any physical activity, and the average sleep duration was 6.9 (± 1.6) hours (Table 1). Sleep quality assessment showed that 20.4% (n = 170) had poor quality, 39% (n = 325) had fair quality, 30.3% (n = 252) had good quality, 7.9% (n = 66) had very good quality, and 2.4% (n = 20) had excellent quality of sleep (Table 1).

Almost one-third (31.6%, n = 267) of the sample reported feeling a state of burnout, according to the WHO criteria. High levels of burnout (i.e., clinical burnout) were found in 54.6% for PB, 50.1% for WB, and 33.2% for CB scores. Scores across all CBI domains were significantly higher among those with suicidal ideation (Table 2).

**Table 2:** Burnout scores according to demographic, work-related, and lifestyle characteristics (n = 844)

	PB score mean ± SD	WB score mean ± SD	CB score mean ± SD
Sex			
Female	53.9 ± 20.3	48.7 ± 20.2	38.8 ± 24.3
Male	44.1 ± 19.3	41.6 ± 18.2	35.8 ± 22.7
p-value	< 0.001	< 0.001	0.158
Ethnicity			
White	50.8 ± 20.4	46.1 ± 19.9	3 7.4 ± 23.8
Nonwhite	55.9 ± 20.2	51.1 ± 19.8	40.8 ± 24.6
p-value	0.002	0.002	0.078
Household income over 5,000 BRL*			
No	56.7 ± 20.7	50.5 ± 21.2	40.9 ± 25.1
Yes	49.1 ± 19.7	45.3 ± 19.0	36.5 ± 23.2
p-value	< 0.001	< 0.001	0.009
Marital status			
No partner (separated/divorced, widowed, single not dating)	53.3 ± 20.8	47.7 ± 19.9	38.7 ± 24.2
Has partner (married/civil union, single but dating)	51.6 ± 20.3	4 7.2 ± 20.1	38.1 ± 23.9
p-value	0.271	0.767	0.748
Has children			
No	54.8 ± 18.9	51.3 ± 18.3	42.9 ± 23.7
Yes	50.5 ± 21.1	45.1 ± 20.6	35.6 ± 23.8
p-value	0.003	< 0.001	< 0.001
Occupation			
Physician	48.1 ± 20.2†‡	45.0 ± 19.2†‡	37.5 ± 23.5†
Nurse	54.6 ± 20.2‡§	49.3 ± 20.5†‡	40.2 ± 23.5‡
Nurse technician	58.3 ± 20.5§	50.8 ± 21.5‡	40.0 ± 27.4‡
Psychologist	46.8 ± 18.5†‡	43.9 ± 18.2†	30.8 ± 21.4†
Others	53.4 ± 199‡§	48.2 ± 19.8†‡	39.9 ± 22.2‡
p-value	< 0.001	0.009	0.036
Workplace: University			
No	52.4 ± 20.5	47.6 ± 20.3	38.7 ± 24.4
Yes	49.9 ± 20.1	45.6 ± 18.1	34.5 ± 20.8
p-value	0.271	0.360	0.104
Workplace: Public hospital			
No	52.1 ± 20.8	46.9 ± 19.9	38.4 ± 23.9
Yes	52.1 ± 19.9	48.0 ± 20.1	38.1 ± 24.3
p-value	0.978	0.403	0.876
Workplace: Private hospital			
No	51.8 ± 20.5	4 7.2 ± 20.3	3 7.9 ± 23.5
Yes	52.9 ± 20.3	47.8 ± 19.3	39.1 ± 25.3
p-value	0.494	0.667	0.524
Workplace: Outpatient primary care			
No	51.0 ± 20.4	46.4 ± 20.2	36.0 ± 24.0
Yes	55.0 ± 20.4	49.9 ± 19.4	44.5 ± 22.9
p-value	0.013	0.021	< 0.001
Workplace: Outpatient mental health			
No	52.2 ± 20.5	4 7.3 ± 20.2	38.5 ± 24.2
Yes	50.6 ± 19.2	4 7.3 ± 15.8	33.4 ± 21.2
p-value	0.628	0.988	0.166
Workplace: Private office			
No	53.7 ± 20.7	48.7 ± 20.3	39.9 ± 24.2
Yes	47.8 ± 19.2	43.8 ± 18.8	33.9 ± 23.2
p-value	< 0.001	0.002	0.001
Assistance COVID-19 patients			
No	47.64 ± 20.31†	42.49 ± 20.11†	35.82 ± 25.53
Yes, indirect assistance	50.39 ± 19.20†	45.90 ± 18.90†	37.81 ± 21.51
Yes, direct assistance	55.49 ± 20.58‡	50.83 ± 19.92‡	39.86 ± 24.26
p-value	< 0.001	< 0.001	0.117
Confirmed COVID-19 diagnosis			
No	51.9 ± 20.4	4 7.2 ± 20.0	38.1 ± 24.0
Yes	55.0 ± 21.0	50.0 ± 19.9	39.9 ± 23.9
p-value	0.297	0.328	0.602
Belonging to high-risk group for COVID-19			
No	50.5 ± 20.5	46.6 ± 19.6	3 7.4 ± 23.7
Yes	56.3 ± 19.8	49.3 ± 21.2	40.5 ± 24.7
p-value	< 0.001	0.086	0.103
Physical activity			
No	54.1 ± 20.4	48.8 ± 19.8	39.1 ± 24.4
Yes	46.1 ± 19.3	43.1 ± 19.8	35.8 ± 22.6
p-value	< 0.001	< 0.001	0.086
Self-perceived/feeling in a state of burnout			
No	43.9 ± 16.7	39.8 ± 16.9	31.1 ± 21.1
Yes	69.7 ± 16.2	63.7 ± 16.2	53.6 ± 22.7
p-value	< 0.001	< 0.001	< 0.001
Childhood abuse or trauma			
No	49.7 ± 20.1	45.4 ± 19.5	36.0 ± 23.3
Yes	5 7.9 ± 19.9	52.3 ± 20.2	43.8 ± 24.8
p-value	< 0.001	< 0.001	< 0.001
History of psychiatric diagnosis			
No	48.5 ± 20.1	43.7 ± 19.9	35.9 ± 23.8
Yes	59.4 ± 19.2	54.7 ± 18.2	42.9 ± 23.7
p-value	< 0.001	< 0.001	< 0.001
Suicidal ideation in the last month			
No	50.4 ± 19.7	46.1 ± 19.5	3 7.0 ± 23.5
Yes	71.2 ± 18.8	61.6 ± 19.1	52.2 ± 24.8
p-value	< 0.001	< 0.001	< 0.001
WEEI-7 total score|| (p-value)	-0.360 (< 0.001)	-0.443 (< 0.001)	-0.405 (< 0.001)
Quality of sleep¶ (p-value)	-0.439 (< 0.001)	-0.389 (< 0.001)	-0.285 (< 0.001)
Age|| (p-value)	-0.228 (< 0.001)	-0.258 (< 0.001)	-0.196 (< 0.001)
Hours of sleep|| (p-value)	-0.196 (< 0.001)	-0.176 (< 0.001)	-0.140 (< 0.001)
Years of profession¶ (p-value)	-0.200 (< 0.001)	-0.202 (< 0.001)	-0.150 (< 0.001)

CB = patient-related burnout; CBI = Copenhagen Burnout Inventory; PB = personal burnout; SD = standard deviation; WB = work-related burnout; WEEI-7 = Work Environment Evaluation Instrument-7.

* For the purposes of this study, 5.22 BRL = 1 USD.

† Groups with this symbol do not present statistically significant differences between them.

‡ Groups with this symbol do not present statistically significant differences between them.

§ Groups with this symbol do not present statistically significant differences between them.

|| Pearson correlation coefficient.

¶ Spearman correlation coefficient.

In terms of COVID-19 exposure, 48.2% (n = 407) were involved in direct care of COVID-19 patients. Frontline professionals were predominantly physicians (52.8%, n = 162), nurses (59.3%, n = 80), and nursing technicians (60.9%, n = 106), compared to psychologists (15.8%, n = 12) and other groups (30.9%, n = 47; p < 0.001). Additionally, 27.6% (n = 233) were not involved in any care of COVID-19 patients, while 24.2% (n = 204) provided such care only indirectly (Table 1). PB and WB scores were higher among frontline HCWs compared to other groups (Table 2).

Among the total sample, 26.9% (n = 227) considered themselves at high risk for COVID-19, with nursing technicians (36.8%, n = 64) perceiving higher risk than physicians (21.5%, n = 66; p = 0.003). Six percent (n = 51) of participants had tested positive for COVID-19, with a higher proportion among nursing technicians (10.3%, n = 18; p = 0.001). Higher PB and WB scores were observed in those perceiving high risk, while scores did not differ for those who tested positive (Table 2).

For frontline professionals, PPE availability significantly impacted CBI scores, with lower scores when PPE was always available. Several factors, such as task coercion, perception of high risk for COVID-19, and maintaining distance from family, were associated with higher CBI scores (Table 3). The proportion of suicidal ideation among frontline HCWs was 7.6%, consistent with the overall sample.

**Table 3:** Frontline HCW burnout scores (n = 407)

Variable	PB score mean ± SD	WB score mean ± SD	CB score mean ± SD
Leaving home/being apart to protect family members while providing care to COVID-19 patients			
No	53.1 ± 21.2	48.6 ± 20.6	38.2 ± 24.5
Yes	59.6 ± 18.6	54.6 ± 18.1	42.8 ± 23.6
p-value	0.002	0.003	0.064
PPE availability			
Not always	60.0 ± 19.5	56.1 ± 18.0	42.5 ± 23.7
Always	51.3 ± 20.4	46.0 ± 20.2	3 7.2 ± 24.3
p-value	< 0.001	< 0.001	0.029
Coercion – Clinical skills beyond training			
No	53.3 ± 20.4	48.4 ± 19.9	3 7.7 ± 23.6
Yes	66.1 ± 18.0	62.5 ± 15.7	50.4 ± 25.0
p-value	< 0.001	< 0.001	< 0.001
Coercion – Unwanted professional activity			
No	51.9 ± 19.9	46.9 ± 18.9	36.3 ± 22.6
Yes	68.1 ± 1 7.5	64.6 ± 16.9	52.4 ± 25.9
p-value	< 0.001	< 0.001	< 0.001
Confirmed COVID-19 diagnosis			
No	55.5 ± 20.6	50.8 ± 19.9	39.7 ± 24.0
Yes	55.3 ± 20.8	51.6 ± 20.5	41.4 ± 2 7 .0
p-value	0.949	0.328	0.602
Belonging to high-risk group for COVID-19			
No	53.4 ± 21.1	49.2 ± 19.7	38.5 ± 24.1
Yes	62.2 ± 1 7 . 2	56.0 ± 19.7	44.1 ± 24.4
p-value	< 0.001	0.003	0.050

CB = patient-related burnout; HCW = health care worker; PB: personal burnout; PPE: personal protective equipment; WB: work-related burnout.

Sociodemographic, personal, clinical, and work-related variables associated with burnout symptoms included being female, providing direct care to COVID-19 patients, and having a history of psychiatric diagnosis. Physical activity correlated negatively with PB and WB scores, while age and higher household income were inversely related to all CBI domains. Childhood trauma and perception of high risk for COVID-19 correlated positively with PB, WB, and CB scores. Weekly working hours correlated positively with WB scores, while a supportive work environment correlated inversely with all CBI domains (Table 4).

**Table 4:** Hierarchical multivariate linear regression analysis to assess independent factors associated with burnout scores

Variable	PB score β/B (95%CI)	p-value	WB score β/B (95%CI)	p-value	CB score β/B (95%CI)	p-value
Block 1 – Demographic						
Female	0.17/8.68 (5.27 to 12.1)	< 0.001	0.13/6.54 (3.17 to 9.91)	< 0.001	0.04/2.40 (-1.75 to 6.55)	0.257
Age	-0.22/-0.42 (-0.54 to -0.30)	< 0.001	-0.26/-0.47 (-0.59 to -0.35)	< 0.001	-0.19/-0.42 (-0.57 to -0.28)	< 0.001
Household income over 5,000 BRL*	-0.13/-5.52 (-8.27 to -2.76)	< 0.001	-0.08/-3.40 (-6.12 to -0.68)	0.014	-0.07/-3.37 (-6.71 to -0.02)	0.048
Block 2 – Childhood trauma	0.15/6.54 (3.67 to 9.41)	< 0.001	0.13/5.57 (2.74 to 8.41)	< 0.001	0.13/6.77 (3.26 to 10.3)	< 0.001
Block 3 – Work, health, and lifestyle						
Direct COVID-19 patient care	0.08/3.12 (0.65 to 5.58)	0.013	0.07/2.83 (0.47 to 5.19)	0.019	-0.01/-0.25 (-3.31 to 2.81)	0.875
Having children	0.02/1.03 (-1.93 to 3.98)	0.496	-0.01/-0.36 (-3.20 to 2.47)	0.801	-0.03/-1.70 (-5.37 to 1.97)	0.363
Hours of work/week	0.06/0.07 (-0.01 to 0.14)	0.068	0.10/0.11 (0.04 to 0.18)	0.002	0.04/0.06 (-0.03 to 0.15)	0.191
High-risk group for COVID-19	0.15/6.78 (3.99 to 9.58)	< 0.001	0.09/4.02 (1.34 to 6.69)	0.003	0.08/4.40 (0.93 to 7.87)	0.013
History of psychiatric diagnosis	0.16/6.69 (4.08 to 9.29)	< 0.001	0.15/6.53 (4.03 to 9.03)	< 0.001	0.04/2.18 (-1.06 to 5.42)	0.1 87
Physically active	-0.11/-5.12 (-7.92 to -2.33)	< 0.001	-0.07/-3.39 (-6.07 to -0.71)	0.013	-0.02/-0.50 (-1.79 to 0.79)	0.448
WEEI-7 total score	-0.29/-0.85 (-1.03 to -0.67)	< 0.001	-0.37/-1.09 (-1.26 to -0.92)	< 0.001	-0.37/-1.29 (-1.51 to -1.07)	< 0.001

B = regression or angular coefficient; CB = patient-related burnout; PB = personal burnout; WB = work-related burnout; WEEI-7 = Work Environment Evaluation Instrument-7; Β = standardized beta coefficient.

* For the purposes of this study, 5.22 BRL = 1 USD.

The hierarchical regression model linked being female, direct assistance of COVID-19 patients, and a history of psychiatric diagnosis to higher PB and WB scores. Physical activity, age, and higher household income correlated inversely with all CBI domains. Childhood trauma and perception of being at high risk of COVID-19 correlated positively with CBI scores. Weekly working hours correlated positively with WB scores, while a supportive work environment (according to WEEI-7 scores) was inversely associated with all CBI domains. These variables also remained significant for clinically significant burnout.

The hierarchical multiple Poisson regression model revealed that childhood trauma and past psychiatric diagnosis significantly increased suicidal ideation likelihood, while a supportive work environment decreased it. These findings remained significant after controlling for sex, age, household income, having children, weekly working hours, perception of being at high risk for COVID-19, and physical exercise (Table 5).

**Table 5:** Hierarchical multivariate Poisson regression to assess independent factors associated with suicidal ideation in the last month

Variable	Suicidal ideation in the last month Exp(b) (95%CI)	p-value
Block 1 – Demographic		
Female gender	0.67 (0.39-1.16)	0.1 51
Age	0.98 (0.96-1.00)	0.076
Household income over 5,000 BRL	0.64 (0.40-1.01)	0.054
Block 2 – Childhood trauma	2.24 (1.43-3.51)	< 0.001
Block 3 – Work, health, and lifestyle		
Direct COVID-19 patient care	1.50 (0.96-2.35)	0.074
Having children	1.15 (0.69-1.91)	0.603
Hours of work/week	1.00 (0.99-1.02)	0.776
High-risk group for COVID-19	1.12 (0.70-1.79)	0.644
History of psychiatric diagnosis	2.28 (1.43-3.63)	< 0.001
Physically active	0.77 (0.43-1.37)	0.771
WEEI-7 total score	0.95 (0.92-0.98)	0.002

Exp(b) = exponential regression coefficient; WEEI-7 - Work Environment Evaluation Instrument-7.

*For the purposes of this study, 5.22 BRL = 1 USD.

## DISCUSSION

More than half of our sample showed elevated levels of burnout, according to their CBI scores. The respondents scored highest in the PB and WB CBI domains, while CB was the lowest. Furthermore, 8.3% of the total sample reported suicidal ideation in the last month. Although our data cannot be used to infer the prevalence of burnout and suicidal ideation among Brazilian HCWs during the COVID-19 pandemic due to the nature of the study design, several risk factors could be identified. Notably, work environment support emerged as the most significant factor associated with burnout (higher β), even when accounting for other individual and occupational variables. This finding is consistent with previous studies conducted by our group, which were based on population samples of psychiatry residents from the Southern region of Brazil.^23,24^ Similarly, the rate of suicidal ideation observed matches those found in other Brazilian studies involving physicians^25^ and medical students,^27^ as well as a meta-analysis^28^ assessing HCW populations.

Other risk factors independently associated with higher levels of burnout in our sample were also found in the burnout literature. These factors were younger age,^6,8,14^ female sex,^6,8,14^ psychiatric diagnosis,^29^ belonging to a high-risk group for COVID-19,^11^ childhood trauma,^29^ lower income,^11^ sedentary lifestyle,^9^ higher workload, and being a frontline HCW.^6,8^ Although we also included HCWs not involved in direct care of COVID-19 patients, the levels of burnout in the overall sample were similar to those reported in studies conducted in LMIC samples composed only of frontline HCWs.^8,14^

It is interesting to mention that the levels of burnout in our study showed a falling gradient from higher to lower level of occupational exposure to COVID-19, which is consistent with trends reported in previous epidemics.^26^ Moreover, as in other studies,^2,8^ providing direct care to patients with COVID-19 was independently associated with PB and WB CBI scores, but not with CB CBI scores, suggesting that patients may only have a minor role in burnout among HCWs in the context of the pandemic. Therefore, the burden associated with a major health crisis seems to be more related to contextual constraints and overwork than to increased demands stemming from patient care itself. As observed in other studies conducted in LMICs,^11,14^ inadequate availability of PPE and being compelled to engage in professional activities one did not feel qualified to do were also linked to higher levels of burnout among frontline HCWs.

The independent association of a history of psychiatric diagnosis and childhood trauma with burnout and suicidal ideation in the last month are in line with the results of a recent study which found that associations between childhood maltreatment and stress from university life were partly mediated by psychiatric morbidity.^29^ Moreover, the association of burnout with lower household income in our study is likely influenced by other burnout risk factors. These factors include nonwhite ethnicity, lower levels of formal education, lower salaries, and decreased levels of autonomy associated with specific categories of HCWs, such as nursing technicians and other technicians included in our sample.

Regarding suicidal ideation rates, HCWs are recognized as a high-risk group for suicide^13,15^ and mental health conditions related to high levels of occupational stress. In fact, occupational stress has been associated with a twofold risk of suicidal ideation in HCWs.^3^ This finding is in line with our results, which showed that CBI scores among HCWs with suicidal ideation were markedly higher than those of HCWs without suicidal ideation. Indeed, a large cohort study of individuals who died by suicide revealed that HCWs had a higher likelihood of facing issues related to their jobs and physical health compared to the general population.^13^

The present study has limitations inherent to its cross-sectional design and reliance on an online convenience sample recruited via the snowball method. This method may have atracted individuals who were already highly engaged, interested in the subject, and had internet access, potentially excluding more vulnerable individuals from the study.^30^ In fact, our sample had a remarkable proportion of individuals with a higher-than-average household income compared to average Brazilians. Therefore, the external validity of these findings needs confirmation in more diverse samples of HCWs and longitudinal studies to assess potential changes over time, as well as to establish probable causality between burnout, suicidal ideation, and the identified associated factors.

These limitations notwithstanding, a notable strength of our study is that it assessed the impact of not only individual but also organizational aspects of the work environment associated with burnout and suicidal ideation during the pandemic, addressing the scarcity of such data from LMICs like Brazil. Moreover, burnout was evaluated using the CBI, a publicly available instrument that underwent thorough cross-cultural adaptation for the target population through a validation study^21^ and is considered a patient-reported outcome measure with sufficient evidence of content validity to assess burnout symptoms.^1^

Moreover, the significant association between perceived work-environment support (WEEI-7) and CBI scores (higher β) in our sample emphasizes the mitigating role of supportive relationships within institutions. This includes leadership style, a collaborative climate, and relationships with the institutions themselves, characterized by shared values and a sense of belonging. These factors significantly impacted HCWs’ levels of burnout and, notably, the presence of suicidal ideation in the last month, even when adjusting for other relevant factors. Thus, they represent a potential target for preventive and corrective interventions to mitigate the risk of mental health issues, even in individuals with other potential risk factors for severe outcomes, such as suicide. Given that burnout and suicidal risk factors among HCWs extend beyond those associated with the COVID-19 pandemic, our results retain relevance even after the complete resolution of this health emergency.

## CONCLUSIONS

In conclusion, despite the aforementioned limitations, our research offers significant theoretical and practical contributions to evidence-based prevention and intervention, which are particularly relevant in countries facing resource constraints and inequalities, such as Brazil.

The impact of workplace support, as underscored in studies conducted in high-income countries,^7^ highlights those modifiable factors that can be addressed through strategies^17^ that promote mental health among HCWs, even in environments constrained by limited availability of financial and human resources.
